# Transcriptome-based identification and characterization of genes responding to imidacloprid in *Myzus persica*e

**DOI:** 10.1038/s41598-019-49922-3

**Published:** 2019-09-16

**Authors:** Jianyu Meng, Xingjiang Chen, Changyu Zhang

**Affiliations:** 1 Guizhou Tobacco Science Research Institute, Guiyang, 550081 China; 2 Guizhou Key Laboratory for Plant Pest Management of Mountain Region, College of Agriculture, Guizhou University, Guiyang, 550025 China

**Keywords:** Transcriptomics, Entomology

## Abstract

*Myzus persica*e is a serious and widespread agricultural pest, against which, imidacloprid remains an effective control measure. However, recent reports indicate that this aphid has evolved and developed resistance to imidacloprid. This study aimed to elucidate the underlying mechanisms and genetic basis of this resistance by conducting comparative transcriptomics studies on both imidacloprid-resistant (IR) and imidacloprid-susceptible (IS) *M*. *persicae*. The comparative analysis identified 252 differentially expressed genes (DEGs) among the IR and IS *M*. *persicae* transcriptomes. These candidate genes included 160 and 92 genes that were down- and up-regulated, respectively, in the imidacloprid-resistant strain. Using functional classification in the GO and KEGG databases, 187 DEGs were assigned to 303 functional subcategories and 100 DEGs were classified into 45 pathway groups. Moreover, several genes were associated with known insecticide targets, cuticle, metabolic processes, and oxidative phosphorylation. Quantitative real-time PCR of 10 DEGs confirmed the trends observed in the RNA sequencing expression profiles. These findings provide a valuable basis for further investigation into the complicated mechanisms of imidacloprid resistance in *M*. *persicae*.

## Introduction

The green peach aphid, *Myzus persicae* (Sulzer), is a widely distributed agricultural pest that has been reported to inflict serious damage on more than 400 plant species, both directly, through phloem feeding, and indirectly, through the transmission of viruses^1^. Over the past several decades, the pest has typically been controlled using synthetic insecticides. However, the excessive use of such insecticides has promoted the development of resistance of *M*. *persica*e to many chemical products, including organophosphates, carbamates and pyrethroids^2^. In contrast, neonicotinoid insecticides, which exhibit high binding affinity to insect nicotinic acetylcholine receptors (nAChRs), remain an effective measure for controlling *M*. *persicae*, owing to their efficacy, long-lasting effects, and harmlessness to mammals^3^. In fact, the first neonicotinoid insecticide, imidacloprid, is still the main product used to control both sucking and biting insect pests and is the world’s most popular insecticide^4,5^. However, as in other types of insecticides, the failure to incorporate insecticide resistance management strategies can increase resistance levels in target pest populations; imidacloprid resistance in *M*. *persicae* has now been reported in the USA, Europe, China, and Japan^6–8^.

The insecticides used in pest management can be considered as environmental stress factors to insect populations. Therefore, it is a common adaptive strategy for pests to develop insecticide resistance^9^. The main mechanisms of neonicotinoid resistance include reduced target-site sensitivity and enhanced metabolic detoxification^10^. A field-evolved instance of imidacloprid resistance in *M*. *persicae* was associated with a single mutation (R81T) in the loop D region of the nAChR β1 subunit, which reduced the binding affinity of nAChR for imidacloprid. This is the first example of field-evolved resistance to imidacloprid that is mediated via a target-site mechanism^4^. However, nAChR mutations have also been reported to play dominant roles in imidacloprid resistance in other insects. In *Nilaparvata lugens*, for example, a target site mutation (Y151S) in the α1 and α3 subunits of nAChRs appears to be responsible for high-level imidacloprid resistance^11^, and in *Musca domestica*, the reduced expression of the α2 subunit of nAChRs has been associated with imidacloprid resistance^12^.

In addition, a number of studies have associated high expression levels of cytochrome P450 genes with neonicotinoids resistance in insects^4^. For example, the overexpression of *CYP6CY3*, *CYP6G1*, *CYP6CM1* and *CYP4C64* has been associated with imidacloprid resistance in *M*. *persicae*, *Drosophila melanogaster* and *Bemisia tabaci*^10,13,14^. An increase in the activities of detoxification enzymes, such as glutathione S-transferases (GSTs) and carboxylesterases, is also known to be associated with imidacloprid resistance in aphids^15^. Although reduced target-site sensitivity and enhanced metabolic detoxification are known to contribute to imidacloprid resistance, it is also possible that both resistance mechanisms and adaptation strategies are complex processes that involve an array of metabolic and genetic factors and that such complex processes are responsible for the development of imidacloprid resistance in *M*. *persicae*.

Global surveys of transcriptional changes in insecticide-treated insects could help elucidate the metabolic and regulatory mechanisms that underlie insecticide resistance. Currently, powerful next-generation RNA sequencing (RNA-Seq) technology can be used to determine the gene expression profiles and, thereby, helping to elucidate the development of insecticide resistance^16–18^. In this study, high-throughput RNA-Seq was used to determine the transcriptome profiles of imidacloprid-resistant (IR) and imidacloprid-susceptible (IS) *M*. *persicae*, with a focus on genes that could provide insight into the mechanisms of physiological adaptation of insects to imidacloprid stress.

## Results

### RNA sequencing

Samples of IS and IR *M*. *persicae* were subjected to Illumina-based RNA-Seq, with three replicates for each strain (IS1, IS2, IS3, IR1, IR2, and IR3). The Illumina sequencing data are shown in Table 1. After filtering, 24,205,298, 24,416,009, and 26,269,490 clean reads were obtained from the IS strain and 28,656,579, 23,803,901, and 23,359,309 clean reads were obtained from the IR strain. The Q20 percentage and GC content of clean reads in the digital gene expression (DGE) libraries ranged from 96.78% to 98.25% and from 39.71% to 40.99%, respectively, and a mean of 92.19% of clean reads was mapped to the *M*. *persicae* genome database.

**Table 1:** Summary of Illumina RNA-sequencing data.

Samples	Raw reads	Clean reads	Q20	GC content	Total mapped	Mapped ratio	Notes
IS1	24,688,350	24,205,298	96.78%	40.55%	44,350,857	91.61%	Replicate 1
IS2	24,914,645	24,416,009	97.67%	39.80%	43,792,242	89.68%	Replicate 2
IS3	26,805,241	26,269,490	98.25%	40.03%	47,999,205	91.36%	Replicate 3
IR1	29,072,436	28,656,579	98.23%	39.55%	53,969,698	94.17%	Replicate 1
IR2	24,481,332	23,803,901	97.66%	39.71%	44,727,119	93.95%	Replicate 2
IR3	24,443,951	23,359,309	97.62%	40.96%	43,139,976	92.34%	Replicate 3

### Differentially expressed genes

**Figure 1:**
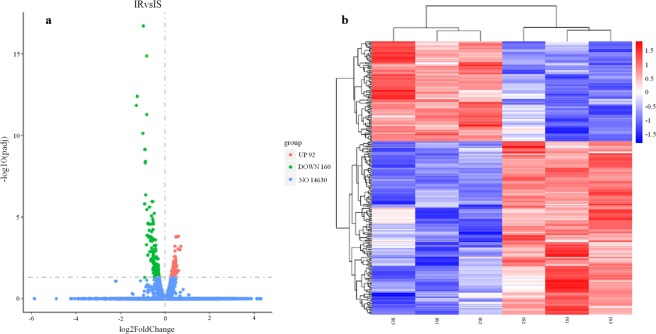
Differentially expressed gene (DEG) analysis of imidacloprid-resistant and imidacloprid-susceptible *Myzus Persicae*. (**a**) Volcano plot of DEGs. Dots represent individual genes. Red dots represent up-regulated genes, and green dots down-regulated genes. Blue dots indicate genes that are not differentially expressed. (**b**) Heatmap analysis of hierarchical clustering of DEGs. Red and blue indicate high and low expression in the imidacloprid-resistant strain, respectively.

Using the DEG-Seq. 2R package, a total of 252 imidacloprid-responsive DEGs were identified in the IS and IR *M*. *persicae* transcriptomes; these included 160 and 92 genes that were down- and up-regulated, respectively, in the IR *M*. *persicae* (Fig. 1, Supplementary Table S1).

### Functional annotation and classification

**Figure 2:**
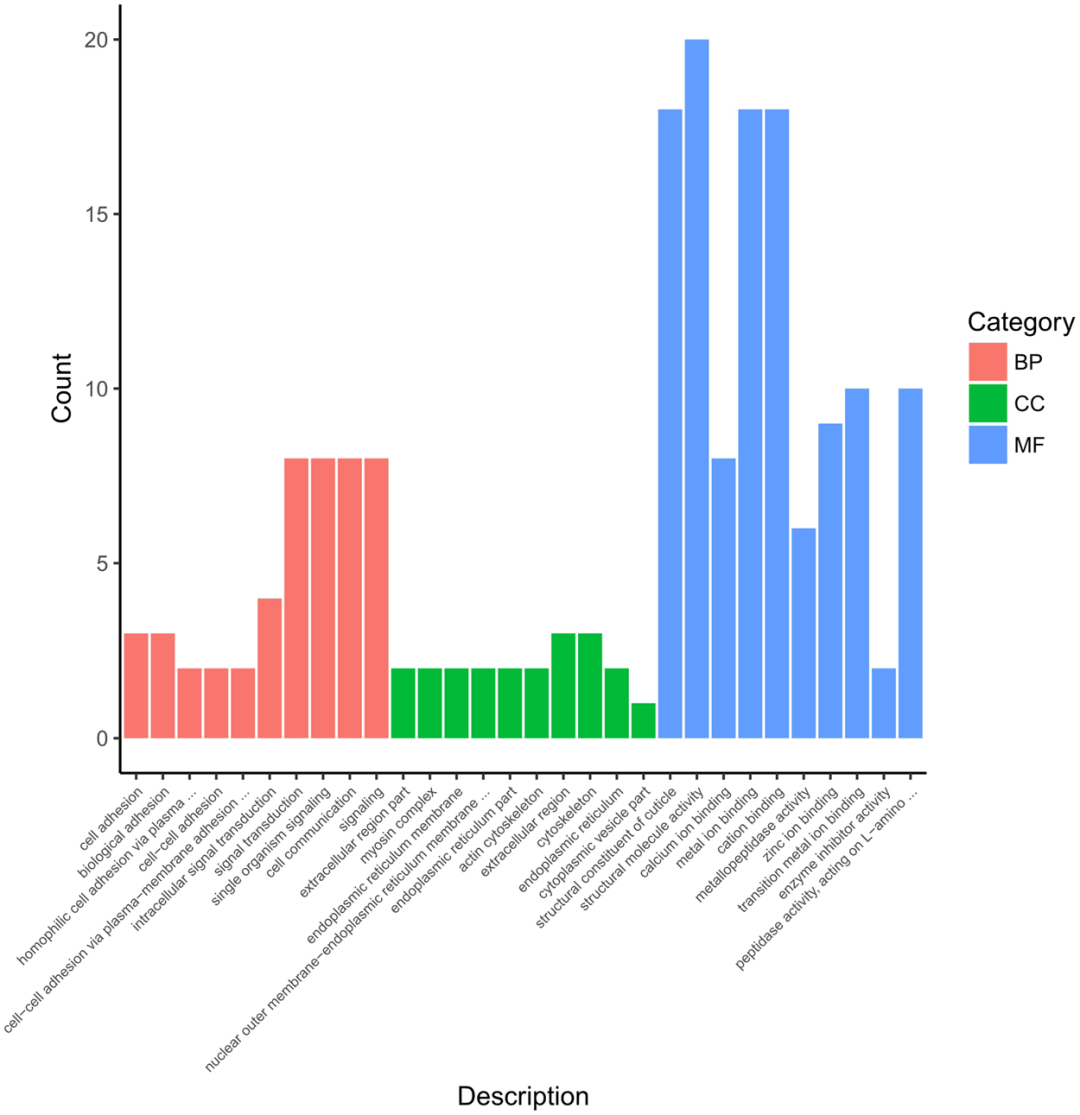
Gene ontology classification of differentially expressed genes. Category of biological process (BP); Category of cellular component (CC); Category of molecular function (MF).

Gene Ontology (GO) indicated that 186 (73.8%) DEGs were assigned to 303 subcategories, including 45 (24.2%) biological process (BP) terms (of which, 88 were significant; corrected *P*-values < 0.05), 36 (19.4%) cellular component (CC) terms, and 105 (56.5%) molecular function (MF) terms (Fig. 2, Supplementary Table S2). Most DEGs in the BP category were putatively attributed to “signal transduction”, “single organism signaling”, “cell communication”, and “signaling”, those in the CC category were attributed to “extracellular region” and “cytoskeleton” and those in the MF category were attributed to “structural molecule activity”, “metal ion binding”, “cation binding”, and “structural constituent of cuticle”.

**Figure 3:**
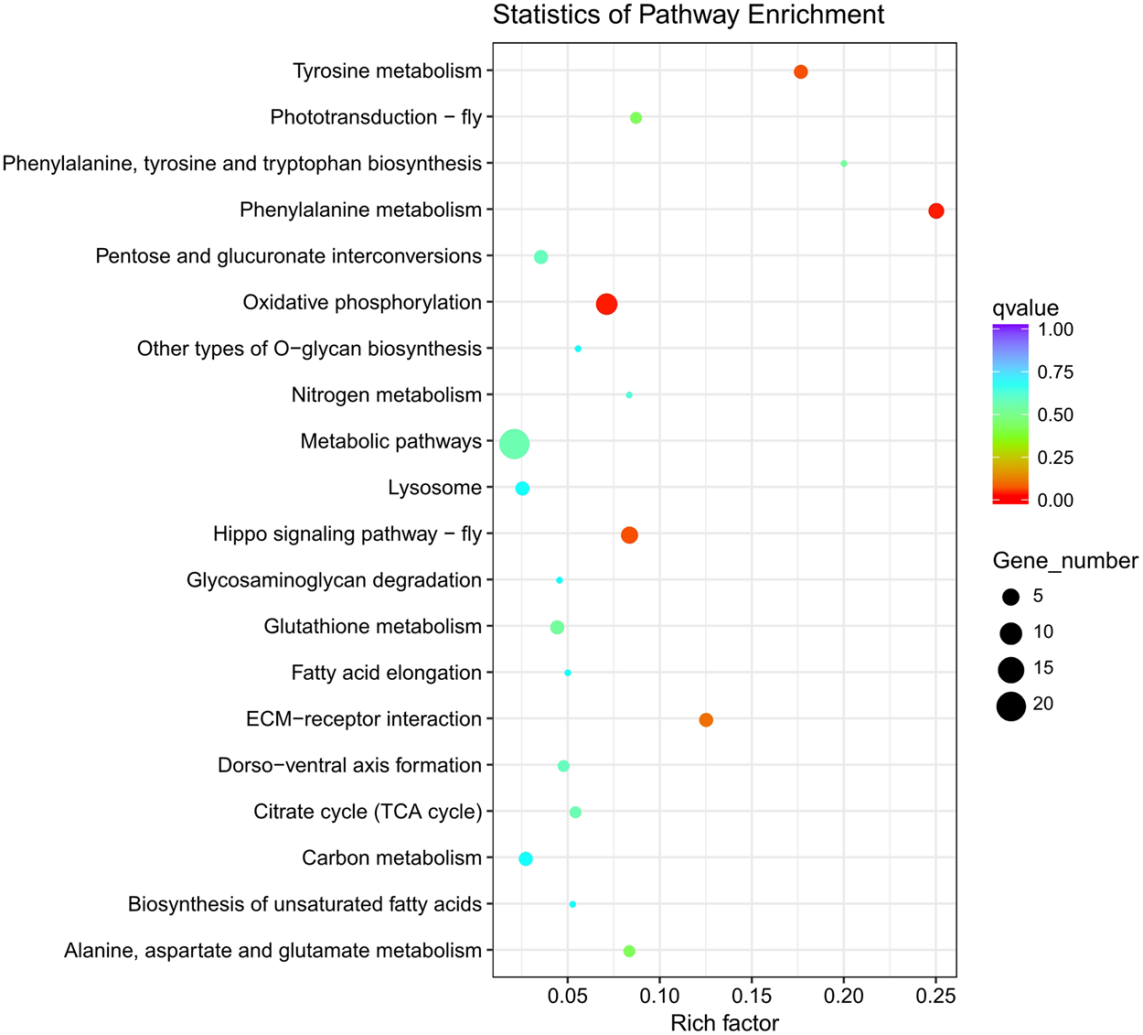
Kyoto Encyclopedia of Genes and Genomes (KEGG) pathway enrichment analysis.

Meanwhile, Kyoto Encyclopedia of Genes and Genomes (KEGG) annotation revealed that 100 DEGs were annotated for *M*. *persicae*. These annotated genes were classified into 45 groups based on the secondary pathway hierarchy (Supplementary Table S3). The major pathways included metabolic pathways (21 proteins), oxidative phosphorylation (9 proteins), Hippo signaling pathway – fly (5 proteins), and phenylalanine metabolism (4 proteins; Fig. 3). In the database of the present study, phenylalanine metabolism (api00360), oxidative phosphorylation (api00190), Hippo signaling pathway – fly (api04391), tyrosine metabolism (api00350), and ECM-receptor interaction (api04512) pathways were found to be with higher corrected *P*-values < 0.05. These annotations establish an invaluable basis for elucidating the specific processes, functions, and pathways involved in the imidacloprid resistance of *M*. *persicae*.

### qRT- PCR validation of DEGs

**Figure 4:**
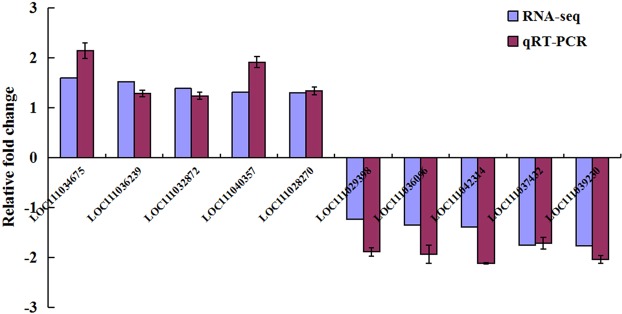
Quantitative real-time PCR (qRT-PCR) validation of the expression of differentially expressed genes identified using RNA-sequencing. The expression levels were normalised to GAPDH, EF1α, and β-actin genes.

qRT-PCR analysis of 10 randomly selected genes confirmed the expression trends observed in the RNA-Seq results (Fig. 4, Supplementary Table S4), thereby, suggesting that the DEG profiles were reliable. Increases in the expression of the selected genes were small, and the biological relevance of these changes is likely minimal.

## Discussion

The green peach aphid, *M*. *persicae*, is an economically important pest that is typically controlled using insecticides. However, the irrational use of imidacloprid has promoted the rapid development of insecticide resistance in many *M*. *persicae* populations, resulting in the failure in controlling the pest^6–8^. Undoubtedly, the development of insecticide resistance is complex and is a common adaptation to insecticide exposure. This study aimed to identify imidacloprid-responsive genes in *M*. *persicae*, to establish a basis for investigating the responses and adaptive physiological changes that contribute to insect resistance.

For insect pests, insecticide exposure can be considered a common environmental stress and the cuticle is the first and main barrier for insects against environmental stresses. As such, cuticular proteins have been reported to play crucial roles in the insecticide resistance and tolerance of a variety of insect species, including *M*. *persicae*, *Lymantria dispar*, *Aphis gossypii* and *Plutella xylostella*^13,16–18^. Indeed, cuticle protein genes (larval cuticle protein A2B/A3A, 111039230/111039229; cuticle protein 111027628, 111034584, 111038352; cuticle protein 12.5/65/38/21/7/3, 111042794/111035272/111032404/111039228, 111039055/111039056/111032178; endocuticle structural glycoprotein SgAbd-2/4/8/9, 111031116/111028315, 111031121/111031114/111041497) were down-regulated in the IR strain of *M*. *persicae*, when compared to the expression levels in the IS strain. In this study, the down-regulation of cuticle protein genes in the IR strain was consistent with previous studies that reported the down-regulation of eight cuticle protein genes in thiamethoxam-resistant *A*. *gossypii*^17^. Such changes in expression suggest that cuticle protein genes contribute to the protection of *M*. *persicae* from the mechanical damage caused by imidacloprid exposure.

The ABC transporters are responsible for the translocation of a variety of substrates (e.g. nutrients, lipids, inorganic ions and xenobiotics) and can be categorised into eight subfamilies from ABCA to ABCH^19^. The ABC transporters present in the blood-brain barrier of insects can protect the nervous system from insecticides^20^ and are reportedly involved in insecticide resistance^21,22^. Indeed, the contribution of ABC transporters to insecticide resistance has been reported for 27 different insecticides including, imidacloprid, pyrethroids, and avermectin^21,23,24^. With regard to imidacloprid, ABC transporters have been reported to enhance its exclusion from the brain and to hinder its access to target sites^24^. Recently, the down-regulation of these genes was reported to be linked to insecticide resistance^22,25^. Similarly, in our study, it was found that ABCG23 (ABCG23, 111040927) was down-regulated in IR *M*. *persicae*, which suggests that the ABC transporter is involved in imidacloprid metabolism and transport.

Glutathione S-transferases (GSTs) are a widespread superfamily of genes that occur in almost all living organisms and participate in a variety of cellular physiological processes, including the detoxification of harmful endobiotic and xenobiotic compounds. Insect GSTs are generally categorised into two main groups: cytosolic and microsomal, based on their cellular location. In insects, most GSTs are cytosolic proteins and are classified as delta, epsilon, omega, sigma, theta, and zeta^26^. In recent years, a number of studies have investigated the correlation between insect GST genes and insecticide resistance. These studies have demonstrated that insect GSTs play important roles in insecticide detoxification and eliminate the oxidative stress caused by insecticide exposure^27^. The up-regulation of GST genes has been associated with insecticide detoxification^28^. However, the down-regulation of GST genes following insecticide exposure has also been reported in several insect species. In *Leptinotarsa decemlineata*, for example, the expression of *LdGSTe4* and *LdGSTe6* was significantly down-regulated after cyhalothrin, fipronil, and endosulfan exposure, and that of *PxGSTd2*, *PxGSTe2*, *PxGSTe5*, *PxGSTo1*, *PxGSTs1*, *PxGSTs2*, and *PxGSTt1* was down-regulated by β-cypermethrin exposure^29^. Similarly, our results indicated that GST (111036096, 111036826) was down-regulated in IR *M*. *persicae*. Bautista *et al*. suggested that this phenomenon may be an adaptive mechanism to insecticide pressure and an energy trade-off strategy to ensure that energy is allocated to the most effective genes responsible for detoxification when stimulated by insecticide exposure^30^. This suggests that GST plays a relatively minor role in the imidacloprid metabolism of *M*. *persicae*. However, because insect GST genes exhibit a variety of expression responses to insecticide exposure^29^, functional studies are needed to examine this hypothesis and identify the specific GST members involved in insecticide detoxification.

Trypsin is a serine protease responsible for digestion; it also contributes to insecticide detoxification^31^. Recently, Zhu *et al*. (2015) reported that the midgut trypsin activity of Bt-resistant *Spodoptera frugiperda* was relatively lower than that of a susceptible strain^32^. Indeed, in our study, trypsin gene (111033016) was also down-regulated in IR *M*. *persicae*. These findings suggest that the expression and function of trypsin are associated with insecticide resistance. However, little is known about the exact role of trypsin in imidacloprid resistance. It has been demonstrated that the lack of midgut trypsin made some insects adapt to insecticide toxins by a mechanism where incomplete or non-activation of the protoxin occurs, and finally induced resistance development^33^.

Mitochondria play critical roles in a variety of cellular processes, among which energy generation is the most critical and which is primarily achieved through coupled oxidative phosphorylation. The electron transport chain (ETC) consists of four macromolecular protein complexes (complex I–IV), that coordinate to maintain mitochondrial inner membrane potential^34^. In this study, the mitochondrial complex-related genes *NADH dehydrogenase* (ETC I, 111029398), *succinate dehydrogenase* (ETC II, 111027244), *cytochrome b-c1 complex* (ETC III, 111038823, 111027246), and *cytochrome c oxidase* (ETC IV, 111034852) were all down-regulated in IR *M*. *persicae*, which indicated that imidacloprid exposure reduced the expression of ETC I, II, III, and IV component genes. It is possible that the respiration and energy production of IR aphids may have been weakened by imidacloprid exposure. There is no evidence that the ETC contributed to the enhanced imidacloprid tolerance, but the apparent alteration in the expression of complex I–IV in IR *M*. *persicae* clearly associates the mitochondrial complex-related genes with imidacloprid resistance. Previous studies have suggested that the overexpression of cytochrome P450s is the primary reason for neonicotinoid resistance^35,36^. Indeed, *CYP6CY3*, which is a cytochrome P450 gene in *M*. *persicae*, has been suggested to play a primary role in the development of insecticide resistance^4,13^. However, the association between mitochondrial complexes and cytochrome P450s with the resistance of *M*. *persicae* to imidacloprid requires further investigation.

In conclusion, this study provides, to our knowledge, the first description of genes related to imidacloprid resistance in *M*. *persicae* using an RNA-Seq approach. The results indicate that the response patterns of aphids are complex during the development of imidacloprid resistance, as demonstrated by changes in the expression of genes involved cuticle structure, binding, metabolic processes, and oxidative phosphorylation. Further investigations are needed to assess the specific roles of these genes in the response of *M*. *persicae* to the stress of insecticide exposure. These findings will provide a basis for investigating the development and mechanisms of insecticide resistance.

## Materials and Methods

### Aphid strains

One IS strain and one IR strain of *M*. *persica*e were used. The IS strain was obtained from tobacco in Guizhou province, China, in 2009, and was subsequently reared in the absence of insecticides. The IR strain was generated from the IS population under continuous imidacloprid selection pressure^37^. In this study, the imidacloprid resistance of the IR strain was ~45-fold greater than that of the IS strain. Both the IS and IR strains were maintained on tobacco plants at 23–25 °C, with a 16-h photoperiod (16 h light, 8 h dark) and relative humidity of 60%.

### RNA isolation

Total RNA was isolated using TRIzol reagent (Invitrogen, CA, USA). The RNA concentration and purity were measured using a NanoPhotometer^®^ spectrophotometer (IMPLEN, CA, USA). The integrity of RNA was confirmed using the Bioanalyzer 2100 system (Agilent, CA, USA).

### Library preparation and sequencing

DGE-Seq was performed using the mRNA isolated from the IS and IR strains, with three biological replicates per strain. Sequencing libraries were constructed using NEBNext^®^ Ultra^TM^ RNA Library Prep Kit for Illumina^®^ (NEB, USA). Briefly, Poly-T oligo-attached magnetic beads were used to purify mRNA, which was then fragmented using divalent cations, and first-strand cDNA was synthesised using random primers and reverse transcriptase, whereas second-strand was synthesised using DNA Polymerase I and RNase H. After 3′-end adenylation, the cDNA fragments were ligated to adapters and then selectively enriched by PCR. The libraries were purified using the AMPure XP system. The quality of the sample libraries was assessed using the Agilent Bioanalyzer 2100 system. Finally, DGE sequencing was implemented using an Illumina HiSeq 2000 instrument.

### Read mapping and expression quantification

Raw reads in fastq format were processed using in-house Perl scripts. The adapter, ploy-N, and low-quality sequences from raw reads were eliminated to obtain clean reads. Q score, GC content, and sequence duplication level were calculated to obtain high-quality clean reads, which were subsequently used for all the downstream analyses. The clean reads were mapped to the *M*. *persica*e genome (GenBank no. GCA_001856785.1), and the expression levels of the genes were calculated using fragments per kilobase per million reads (FPKM) values^38^.

### Differentially expressed gene (DEG) analysis and annotation

Differential expression analysis was performed using DESeq^39^, and the Benjamini and Hochberg’s method was used to adjust the resulting *P*-values, to minimise the false detection rate (FDR). Genes were identified as differentially expressed (i.e. DEGs) if the adjusted *P*-value was <0.05^40^. The functional annotation and classification of the genes were performed using the GO database, and biological pathway annotations were obtained using the KEGG database.

### Quantitative real-time PCR analysis

Quantitative real-time PCR (qRT-PCR) analysis was performed to validate the expression profiles of 10 randomly selected DEGs and three internal controls, namely the elongation factor 1α (EF1α), β-actin, and glyceraldehyde-3-phosphate dehydrogenase (GAPDH) genes. The primers used in qRT-PCR are summarized in Table 2. qRT-PCR reactions were performed using a SYBR Premix DimerEraser Kit (Takara, Dalian, China) on an ABI 7500 system (ABI, CA, USA). All qRT-PCR experiments were performed in triplicate using independent samples. The expression levels were determined by the 2^−ΔΔCt^ method^41^, using the geometric mean of three selected internal control genes for normalisation.

**Table 2:** Primers used for qRT-PCR validation of differentially expressed genes.

Gene name	Forward primer (5′-3′)	Reverse primer (5′-3′)	Gene description
LOC111034675	5’TGCGGGAGGTGTGAGAGCTG	TCGCCGTTTTTCAATGTATCG	maltase A3-like
LOC111036239	CGCGGTACATGAATTGCACAACTG	ACGCAATGTCGAAGAACGGTATC	aspartate aminotransferase, cytoplasmic-like
LOC111032872	CCGCGTGAGGATATGTGTTGAC	ACGGCCAGAGGACACACGATG	protein O-linked-mannose beta-1, 2-N-acetylglucosaminyltransferase 1-like
LOC111040357	GAGCCAAGAAAATGCAGATGAATAC	TCCGCATGAATGAGACCCAAATC	homeodomain-interacting protein kinase 2
LOC111028270	TCCCGGGTTTATCGTGGCAAG	CCCAACAACATGAGCAACAAATAAC	serine/threonine-protein kinase tousled-like 2
LOC111029398	CGCCCGATGCCATTAGTTCAAC	TGGCATTCAAGTCATCTGTCTCATC	NADH dehydrogenase [ubiquinone] 1 beta subcomplex subunit 11,mitochondrial
LOC111036096	GGCAGCATACAAACTCACTTACTTC	AGGGCATTTTGGGCTTGATTG	glutathione S-transferase-like
LOC111042314	GCCGCCGAACAGTCTGCAAAAC	AGCGGCCAGGTAGGGTGAAG	serine/arginine-rich splicing factor 8-like
LOC111037432	CGCCAAAACATCAACAATCAACAAG	GGTTGGCGTGGTTGTTAAGATTTG	GATA zinc finger domain-containing protein 14-like
LOC111039230	GCGACGACGTGACCGGTTACTAC	TGGCGGCCTTAGCGACGATC	larval cuticle protein A2B-like
EF1α	CCGATGTCTATGTCTGCTAAGG	CATGATTTGAGCCTCGCCAA	
β-actin	CGGTTCAAAAACCCAAACCAG	TGGTGATGATTCCCGTGTTC	
GAPDH	GCGGTTTCGACGTGTCAGTTTG	CCGGAGCCCACAATGCACAC	

## Supplementary information

Supplementary Table S1

Supplementary Table S2

Supplementary Table S3

Supplementary Table S4

## Data Availability

The RNA-Seq raw data were deposited in the NCBI Sequence Read Archive (SRA) with the accession number PRJNA558181.
